# Mitigation of AFB_1_-Related Toxic Damage to the Intestinal Epithelium in Broiler Chickens Consumed a Yeast Cell Wall Fraction

**DOI:** 10.3389/fvets.2021.677965

**Published:** 2021-07-26

**Authors:** Juan Omar Hernández-Ramírez, Rubén Merino-Guzmán, Guillermo Téllez-Isaías, Alma Vázquez-Durán, Abraham Méndez-Albores

**Affiliations:** ^1^Unidad de Investigación Multidisciplinaria L14 (Alimentos, Micotoxinas, y Micotoxicosis), Facultad de Estudios Superiores Cuautitlán, Universidad Nacional Autónoma de México, Mexico City, Mexico; ^2^Departamento de Medicina y Zootecnia de Aves, Facultad de Medicina Veterinaria y Zootecnia, Universidad Nacional Autónoma de Mexico, Mexico City, Mexico; ^3^Department of Poultry Science, University of Arkansas, Fayetteville, AR, United States

**Keywords:** broilers, AFB1, yeast cell wall, intestinal permeability, histomorphology

## Abstract

*In vivo* experiments were conducted to evaluate the effectiveness of a yeast cell wall fraction (YCW) to reduce the negative impact of aflatoxin B_1_ (AFB_1_) to the intestinal epithelium in broiler chickens. Zeta potential (ζ-potential), point of zero charge (pH_pzc_), Fourier transform infrared spectroscopy (FTIR), and scanning electron microscopy (SEM) techniques were used to characterize the YCW. Two hundred one-day-old male Ross 308 broiler chickens were randomly allocated into four treatments: (1) control, chickens fed an AFB_1_-free diet; (2) AF, chickens feed an AFB_1_-contaminated diet (500 ng AFB_1_/g); (3) YCW, chickens fed an AFB_1_-free diet + 0.05% YCW; and (4) AF + YCW, chickens fed an AFB_1_-contaminated diet (500 ng AFB_1_/g) + 0.05% YCW. At the end of the 21-day feeding period, fluorescein isothiocyanate dextran (FITC-d) was administered to chicks by oral gavage to evaluate gastrointestinal leakage. Blood and duodenum samples were collected to assess serum biochemistry and histomorphology, respectively. Compared to the control group, chicks of the AF group significantly diminished weight gain (WG) and average daily feed intake (ADFI), and increased feed conversion ratio (FCR), mortality rate (MR), and intestinal lesion scores (*p* < 0.05). Alterations in some serum biochemical parameters, and damage to the intestinal integrity were also evident in the AF-intoxicated birds. YCW supplementation improved WG and FCR and increased villus height, villus area, crypt depth, and the number of goblet cells in villi. The effects of YCW on growth performance were not significant in chicks of the AF + YCW group; however, the treatment decreased MR and significantly ameliorated some biochemical and histomorphological alterations. The beneficial effect of YCW was more evident in promoting gut health since chickens of the AF + YCW group presented a significant reduction in serum FITC-d concentration. This positive effect was mainly related to the changes in negative charges of YCW due to changes in pH, the net negative surface charge above the pH_pzc_, the higher quantities of negative charged functional groups on the YCW surface, and its ability to form large aggregates. From these results, it can be concluded that YCW at low supplementation level can partially protect broilers' intestinal health from chronic exposure to AFB_1_.

## Introduction

Filamentous fungi, particularly *Aspergillus, Fusarium*, and *Penicillium*, are capable of forming secondary metabolites known as mycotoxins. Among the many toxic metabolites identified, some of them are potent carcinogens, which can provoke acute or chronic intoxications in both humans and animals. In agricultural commodities, the most frequently encountered mycotoxins are aflatoxins, ochratoxin A, patulin, fumonisins, trichothecenes (deoxynivalenol, T-2 toxin, and HT-2), and zearalenone (1). Some mycotoxigenic fungi can produce more than one toxin, and some mycotoxins are synthesized by multiple fungal species (2). In comparison with other mycotoxins, the safety level for aflatoxins in poultry feedstuffs is low; as a result, poultry feed is always at risk of contamination with aflatoxins, which are frequently found in maize destined for animal feed. When toxigenic *Aspergillus flavus, Aspergillus parasiticus*, or *Aspergillus nomius* isolates grow in poultry feedstuffs, they can synthesize a variety of toxic secondary metabolites, including aflatoxin B_1_ (AFB_1_), aflatoxin B_2_ (AFB_2_), aflatoxin G_1_ (AFG_1_), and aflatoxin G_2_ (AFG_2_). As a result, the accumulation of these toxic metabolites in animal tissues may result in an indirect exposure to humans by consuming the contaminated products such as meat or eggs.

Notwithstanding attempts to monitor fungal and mycotoxin contamination, both developing and developed countries have confirmed widespread contamination. To detoxify mycotoxin-contaminated feeds and feedstuffs, various methods have been proposed based on physical, chemical, and biological approaches. Detoxifying agents are substances that may reduce mycotoxin contamination in feed by suppressing or reducing their absorption, promoting their excretion, or changing their mode of action. These substances—called mycotoxin detoxifiers—are added to animals' diet (poultry, swine, and cattle) to minimize toxin absorption and dissemination to blood and target organs. Based on their mode of action, mycotoxin detoxifiers can bind, inactivate, degrade, or transform mycotoxins into less toxic substances. Activated charcoal, hydrated sodium calcium aluminosilicates, polymers, zeolites, agro-waste materials, yeast, and yeast products are examples of adsorbent materials that can be utilized to reduce the toxic effects of various mycotoxins (2–4).

Yeast cell wall (YCW), mainly composed of polysaccharides (mannans and glucans), proteins, and lipids, possesses a variety of adsorption sites, with different mechanisms of adsorption including hydrogen bonding, hydrophobic interactions, and ionic interactions (5). Therefore, YCW could be an alternative over conventional adsorbent materials to bind a wide variety of mycotoxins (6), without reducing nutrient bioavailability or causing negative environmental impacts. While many studies have been conducted to show that YCW can improve broiler performance and intestinal health when challenged with aflatoxins alone or in combination with pathogens (7, 8), just a few studies have looked into the pathways that lead to the formation of complexes involving mycotoxins and YCW components, where some chemical structures such as (1 → 3)-β-D-glucan or (1 → 6)-β-D-glucan play a significant role during the binding process (9–11). Currently, there is a lack of general knowledge about the application of ζ-potential, point of zero charge, Fourier transform infrared spectroscopy, and scanning electron microscopy techniques to characterize the YWC and to understand the interaction between the functional groups present on the YCW surface and the AFB_1_ molecule. Consequently, this research aimed to describe and evaluate the effectiveness of a commercial YCW fraction's low content to reduce the negative impact of AFB_1_ on the intestinal epithelium in broiler chickens.

## Materials and Methods

### Yeast Cell Walls

A premium yeast cell wall fraction (YCW) from *Saccharomyces cerevisiae* (SafMannan) was kindly provided by Phileo Lesaffre Animal Care (Lesaffre Iberica S.A., Valladolid, Spain). The chemical composition of the commercial YCW fraction according to the manufacturer is shown in Table 1.

**Table 1:** Chemical composition of the yeast cell wall fraction (YCW) according to manufacturer.

**Chemical composition**	**(%)**	**Chemical composition**	**(%)**
Dry matter	97–98	Ash	3–5
Protein	14–17	β-Glucans	24–26
Fat	20–22	Mannans	22–24
Phosphorous	1–2	Glucan to mannan ratio	1.1

### YCW Characterizations

#### Zeta Potential (ζ-Potential)

The electrophoretic mobility measurement and conversion to ζ-potential were made using the ZetaSizer Pro (Malvern Instruments, Worcestershire, UK) following the methodology of Ramales-Valderrama et al. (12). All determinations were done at room temperature by diluting 500 μL of the YCW suspension (0.05% w/v) in 5 ml deionized water. Quintuplicates were evaluated, and each measurement included 30 runs to find a stable reading. Samples were evaluated at three different pH values simulating the poultry gastrointestinal tract's *in vivo* conditions (proventriculus, pH 2; crop, pH 5; and intestine, pH 7).

#### Point of Zero Charge (pH_pzc_)

The pH_pzc_ was determined following the approach used by Zavala-Franco et al. (13). Briefly, 50 mL of distilled water was adjusted to different pH values (2, 4, 6, 8, 10, and 12) by adding 0.1 M hydrochloric acid or 0.1 M sodium hydroxide. The solutions were added into flasks containing preweighed YCW (25 mg) and stirred (250 rpm) at room temperature for 195 min. The final pH (pH_f_) of the suspension was determined, and the pH difference (ΔpH) was computed. All pH measurements were accomplished using a glass electrode (Conductronic PC-45, Puebla, Mexico). Finally, ΔpH was plotted against the initial pH (pH_i_), and the point where the line intersects the *x*-axis gave the pH_pzc_. All determinations were performed in quintuplicate.

#### Fourier Transform Infrared Spectroscopy

Functional groups of the YCW were characterized using a Fourier transform infrared spectroscopy (FTIR) Frontier SP8000 spectrophotometer (Perkin Elmer, Waltham, MA, USA) accessorized with an attenuated total reflection (ATR) accessory (DuraSamplIR II, Smiths Detection, Warrington, UK). Quintuplicate samples (25 mg) were placed on the ATR diamond crystal, and the spectra were recorded in transmittance mode over the range of 4,000–500 cm^−1^ at a resolution of 4 cm^−1^ by coadding 32 scans. The background spectrum of air was subtracted from all the spectra. Additionally, a section of the spectra (the polysaccharide absorbing region, which reveals component structures of mannans and glucans) was baseline corrected, and the resultant FTIR spectrum was further analyzed. The peak areas of the main bands (carbohydrate, protein, and lipid) were computed using the Spectrum 10.4.2 software.

#### Scanning Electron Microscopy

The morphology and microstructure of the YCW were scrutinized using an InTouch Scope scanning electron microscope (SEM) (JEOL, JSM-6012LA, Tokyo, Japan). To enhance electron conductivity and image quality, samples were coated with a thin gold layer using an electric sputter coater (Denton Vacuum Inc., Desk V HP, Moorestown, NJ, USA) operated at 7 mA for 3 min. Microscopy analysis (×250) was performed in the secondary electron imaging mode (SEI mode) with an accelerating voltage of 15 kV at a 17-mm effective working distance.

### *In vivo* Experiments

#### Animal Ethics

The aflatoxin challenge protocol was approved by the Internal Committee for Care and Use of Experimental Animals of the Postgraduate Program in Animal Production and Health Sciences of the National Autonomous University of Mexico. Ethical approval code: CICUAE-C20_5.

#### Aflatoxin B_1_ Production and Preparation of the AFB_1_-Contaminated Diet

Aflatoxins (AFB_1_ and AFB_2_) were produced in maize according to the methodology of Méndez-Albores et al. (14) using a highly toxigenic strain of *A. flavus* (Code UNIGRAS-1231, Culture Collection of the Grain and Seed Research Unit of the National Autonomous University of Mexico). The highly contaminated maize kernels (14,000 ng AFB_1_/g) were milled and subsequently mixed in a starter feed formulated to approximate broiler chickens' nutritional requirements (Supplementary Table 1) as recommended by the National Research Council (15). Contamination was performed in batches of 15 kg using 36 g of the aflatoxin-contaminated maize meal per kilogram of feed. Subsequently, the aflatoxin-contaminated poultry feed was mixed for 15 min in a Ribbon Blender Mixer (Molinos Pulvex model MH-7050, Mexico City, Mexico) to ensure proper distribution of the toxins. The adsorbent material was also included in the feed.

#### Aflatoxin B_1_ Quantification

The aflatoxin content in the feed was estimated by immunoaffinity column clean-up and liquid chromatography with fluorescence detection. Briefly, aflatoxins were cleaned up using immunoaffinity columns Vicam Afla B (Watertown, MA, USA) and the eluate used for ultraperformance liquid chromatography (UPLC) analysis. A modified method previously described by Jardon-Xicotencatl et al. (16) was used. A mobile phase of water/methanol/acetonitrile (64:18:18) was used on an ACQUITY UPLC BEH C18 column (2.1 × 100 mm, 1.7 μm). The mobile phase was pumped at 0.7 mL/min by a quaternary solvent manager. The aflatoxins eluted in the order of AFB_2_ and AFB_1_ at 1.57 and 2.00 min, respectively (Supplementary Figure 1). Detection was via an UPLC-optimized fluorescence detector (Waters, Milford, MA, USA) programmed to detect aflatoxins at 365 nm excitation and 429 nm emission. The estimated detection limits were 0.6 and 2.0 ng/kg for AFB_2_ and AFB_1_, respectively. Finally, the AFB_1_ concentration was calculated using a standard reference (AFB_1_; CAS number, 1162-65-8, Merck KGaA, Darmstadt, Germany) with a calibration curve. All determinations were done in quintuplicate. The UPLC analysis revealed the presence of AFB_1_ (500 ± 21 ng/g feed) and traces of AFB_2_ (43 ± 7 ng/g feed). Since AFB_2_ is up to 200-fold less toxic than AFB_1_ (14), in this research, the presence of AFB_2_ was considered insignificant.

#### Experimental Birds and Housing

A total of 200 1-day-old male broilers (Ross 308) were purchased from a local hatchery. Birds were randomly distributed in four pens at the Poultry Research Station of the National Autonomous University of Mexico. Five replicates of 10 birds (*n* = 50 per treatment) were grouped as follows: (1) control, chickens fed an AFB_1_-free diet; (2) AF, chickens feed an AFB_1_-contaminated diet (500 ng AFB_1_/g); (3) YCW, chickens fed an AFB_1_-free diet + 0.05% YCW (the minimum manufacturers' recommended inclusion rate); and (4) AF + YCW, chickens feed an AFB_1_-contaminated diet (500 ng AFB_1_/g) + 0.05% YCW. Chicks were maintained at an age-appropriate temperature and given *ad libitum* access to diets and water during the 21-day study. Twice a day, birds were monitored for general health.

#### Collection of Samples and Measurements

Birds and feed were weighed weekly, and feed efficiency was adjusted for mortality. At 21 days of age, blood samples were collected from 15 randomly selected broilers from each treatment (three birds per replicate) and serum prepared. The following analyses were accomplished spectrophotometrically using commercially available kits (BioSystems, Barcelona, Spain): total protein (code 11500), albumin (code 11547), glucose (code 12503), cholesterol (code 11505), and the enzymatic activities of aspartate aminotransferase (AST, code 11531) and alanine aminotransferase (ALT, code 21533). The bled chickens were then euthanized by CO_2_ inhalation, and segments of duodenum (2 cm in length) taken from the gizzard outlet to the end of the pancreatic loop were carefully excised, rinsed three times with cold saline, and fixed in 10% neutral-buffered formalin for 48 h. The paraffin-embedded tissue samples were cut into 4-μm thick sections and stained with hematoxylin and eosin (H&E). Photomicrographs were acquired using an ICC50W camera associated with a Leica DM2500 microscope. The variables measured were the following: villus height (measured from the top of the villus to the upper part of the lamina propria), villus width (taken at the central part of the villus), crypt depth (measured from the base up to the region of transition between the crypt and villus), villus area (villus height × villus width), and the goblet cell number along the villi membrane, which were counted along 500 μm of each villus surface. The ImageJ 1.52v software was used for morphometric measurements. In each treatment, 60 measurements were taken per variable.

#### Serum Determination of Fluorescein Isothiocyanate Dextran Leakage

Fluorescein isothiocyanate dextran (3–5 kDa, Merck KGaA, Darmstadt, Germany) was used as a probe to measure gut mucosal barrier integrity. Following the methodology of Baxter et al. (17), 1 h before euthanizing the chickens, 15 randomly selected broilers of each group (three per replicate) were orally gavaged with fluorescein isothiocyanate dextran (FITC-d) (8.32 mg/kg of body weight). The concentration of FITC-d was fluorometrically estimated in diluted sera as described by Hernández-Ramírez et al. (18). Sera from birds without FTIC-d treatment were used as controls.

### Experimental Design and Statistical Analysis

Data were analyzed as a completely randomized design using the one-way ANOVA procedure of the Statistical Analysis System software (19). The replicate pens were the experimental units for the analysis, and means were separated using the Tukey procedure at *p* < 0.05 level of significance.

## Results

### Characterizations of the YCW Fraction

**Figure 1:**
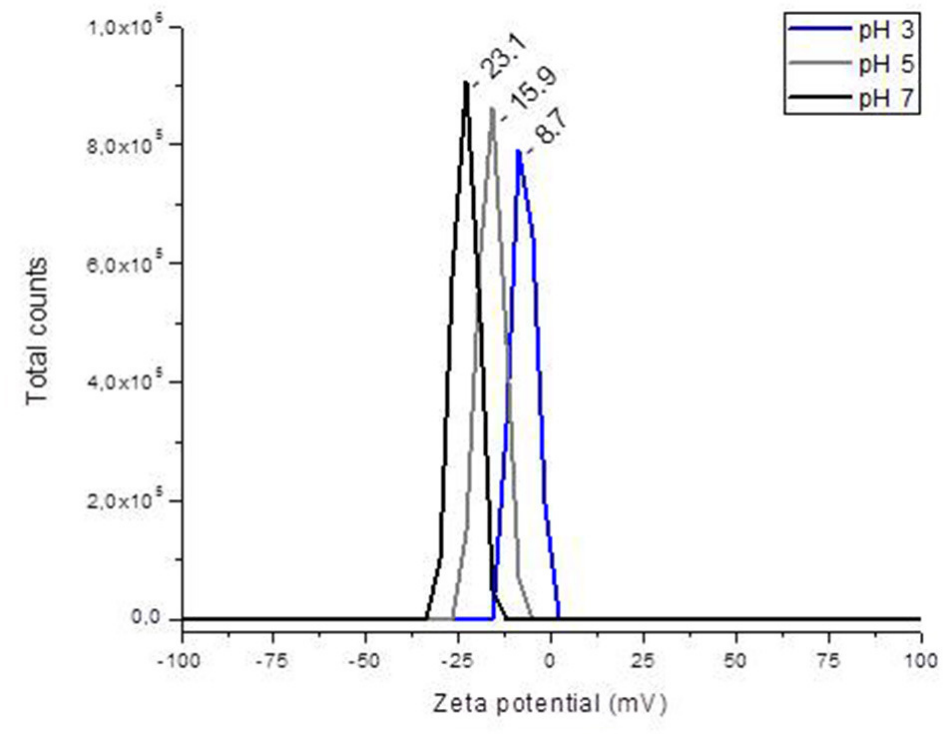
The changes in zeta potential of the yeast cell wall fraction (YCW) with different pH.

**Figure 2:**
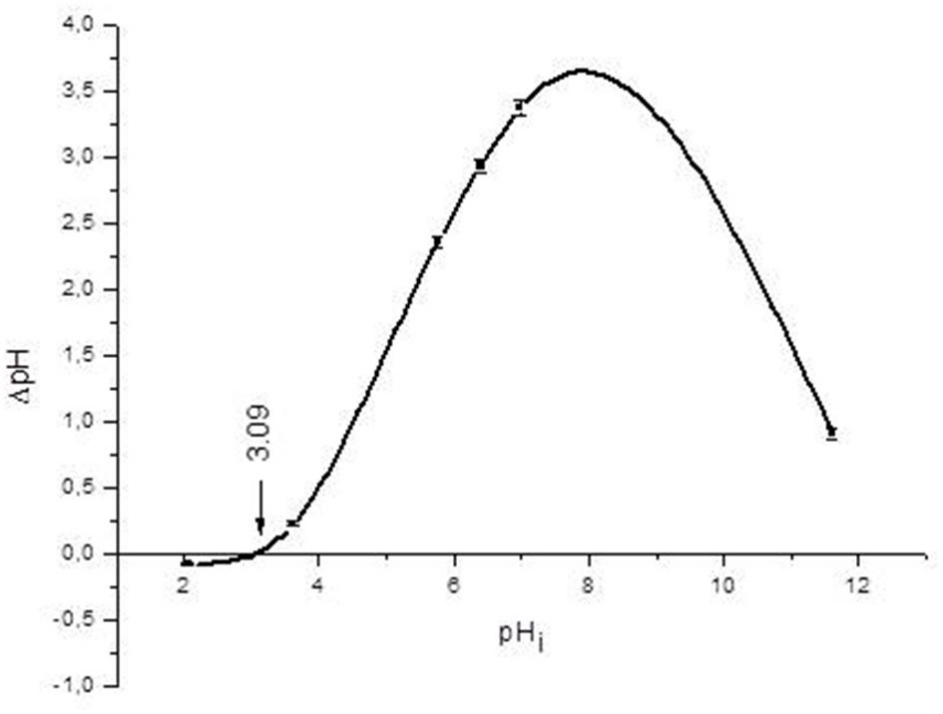
Point of zero charge (pH_pzc_) of the yeast cell wall fraction (YCW). Mean values ± standard error.

Figure 1 shows the relationship between ζ-potential and pH. In general, the YCW product had a negative ζ-potential; however, as the pH value increased (from 2 to 7), more negative ζ-potential values were observed. A ζ-potential value of −8.7 ± 1.3 mV was registered at pH 2; however, at pH 5 and 7, the YCW preparation presented ζ-potential values of −15.9 ± 2.5 mV and −23.1 ± 2.1 mV, respectively. The point of zero charge (pH_pzc_) was determined by plotting ΔpH against the initial pH using the immersion technique. Figure 2 shows the pH_pzc_ of the YCW preparation. In the pH_pzc_ graphic, the curve intersects the *x*-axis at pH 3.09, suggesting that the surface charge is zero at this particular pH. In other words, the charge of the positive surface sites is equal to that of the negative ones.

**Figure 3:**
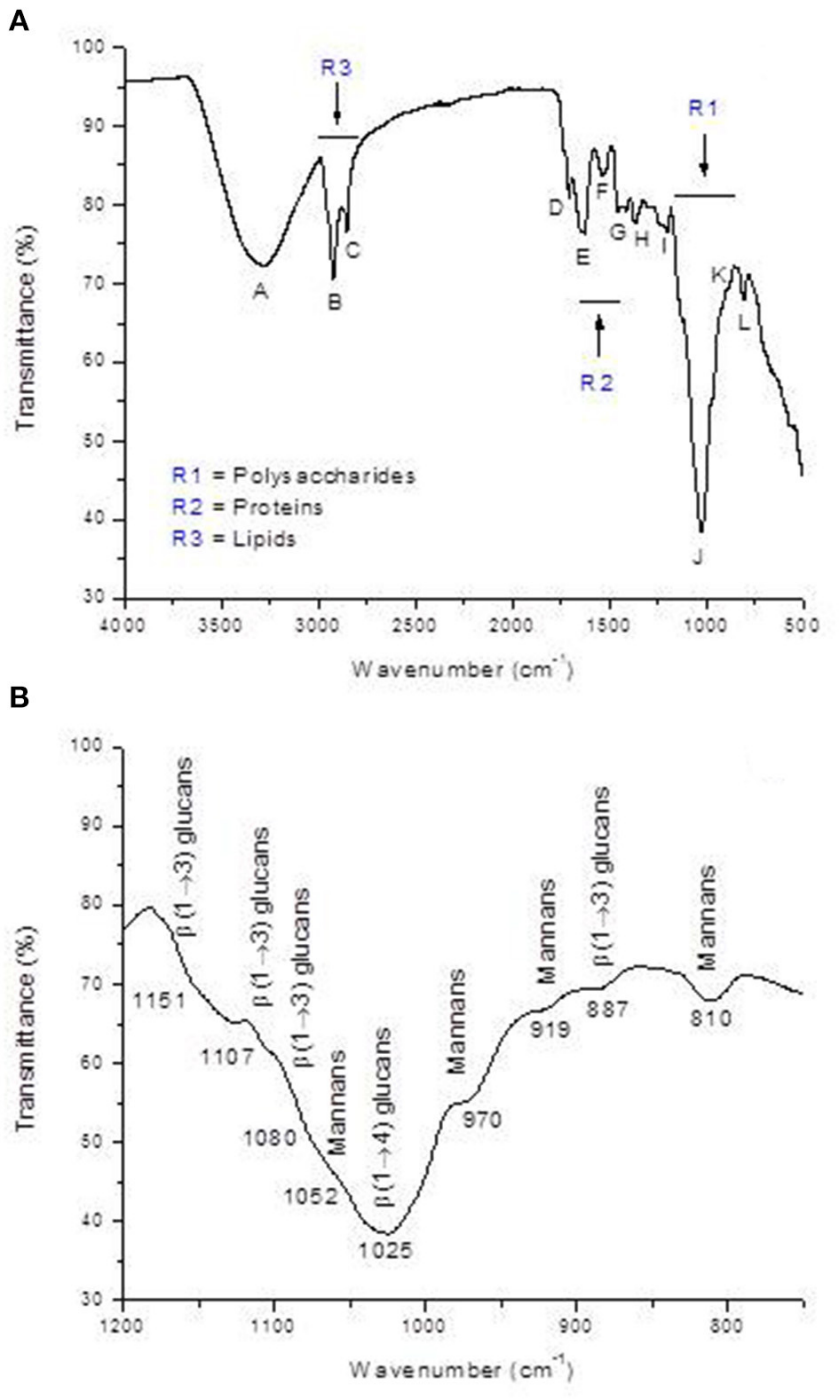
**(A)** Representative FTIR spectrum of the yeast cell wall fraction (YCW), and **(B)** baseline corrected FTIR spectrum of the polysaccharide absorbing region (R1 = 1200-750 cm^−1^).

Furthermore, the FTIR spectrum was acquired to identify the specific functional groups present on the YCW product's surface. The representative FTIR spectrum and the baseline-corrected spectrum of the polysaccharide absorbing region (1,200–750 cm^−1^) are shown in Figure 3. From the spectrum, three main regions corresponding to polysaccharides (R1 = 750–1,200 cm^−1^), proteins (R2 = 1,400–1,650 cm^−1^), and lipids (R3 = 2,800–3,000 cm^−1^) can be clearly distinguished (Figure 3, profile A). Main absorptions characteristic of polysaccharides are those related to O–H stretching (3,279 cm^−1^), β-anomeric carbons of β-glucans (1,369 cm^−1^), C–O stretching and C–OH wagging (1,202 and 1,025 cm^−1^), β-anomeric carbons of β(1 → 3) glucans (887 cm^−1^), and mannans (810 cm^−1^). Characteristic N–H vibrations of proteins were observed at 3,279 cm^−1^ (overlapped by O–H vibrations) and at 1,629 and 1,532 cm^−1^, which were associated with the amide I and amide II bands, respectively. Finally, C–H stretching bands of lipids were located at approximately 2,922 and 2,849 cm^−1^, respectively. These bands were also overlapped by the C–H stretching of glucans (Figure 3, profile A). Moreover, Figure 3 (profile B) depicts the baseline-corrected FTIR spectrum of the carbohydrate absorbing region (frequency range, 1,200–750 cm^−1^). As can be seen from the spectral magnification, the absorptions at 810, 919, 970, and 1,052 cm^−1^ characterize mannans. Additionally, the bands at 887, 1,080, 1,107, and 1,151 cm^−1^ can be assigned to β(1 → 3) glucans. Finally, the broad absorption band at 1,025 cm^−1^ is generally associated with the presence of β(1 → 4) glucans. The assignments of the main vibrational bands are summarized in Table 2. Furthermore, as a useful indicator of the three main components' ratio, the total area corresponding to polysaccharide, protein, and lipid regions was computed using the Spectrum 10.4.2 software. The results show the highest intensity in the polysaccharide region, followed by lipid and protein regions, respectively. In general, the polysaccharide was shown approximately 3.9-fold higher than lipid, and the ratio of polysaccharide to protein was 7.8-fold higher. These results are consistent with the chemical composition of the YCW product shown in Table 1.

**Table 2:** Band assignments of the primary vibrational frequencies in the yeast cell wall fraction (YCW).

**Band**	**Wavenumber (cm^**−1**^)**	**Functional group or commonly assigned compound**
A	3,279	O–H and N–H stretching vibrations (carbohydrate and protein)
B	2,922	CH_2_ antisymmetric stretching (lipids)
C	2,849	C–CH_3_ symmetric stretching (lipids)
D	1,711	C=O stretching (phospholipid esters)
E	1,629	Amide I (N–H bending and C=O stretching)
F	1,532	Amide II (C–N stretching and N–H bending)
G	1,455	OH bending vibration in carboxylic acids
H	1,369	β-anomeric carbons (β-glucans)
I	1,202	C–O stretching, C–OH wagging, twisting, and rocking (carbohydrates)
J	1,025	C–O stretching (carbohydrates)
K	887	β-anomeric carbons β(1 → 3)-glucans
L	810	Mannans (C–O–C, C–C, and C–OH stretching of pyranose ring)

**Figure 4:**
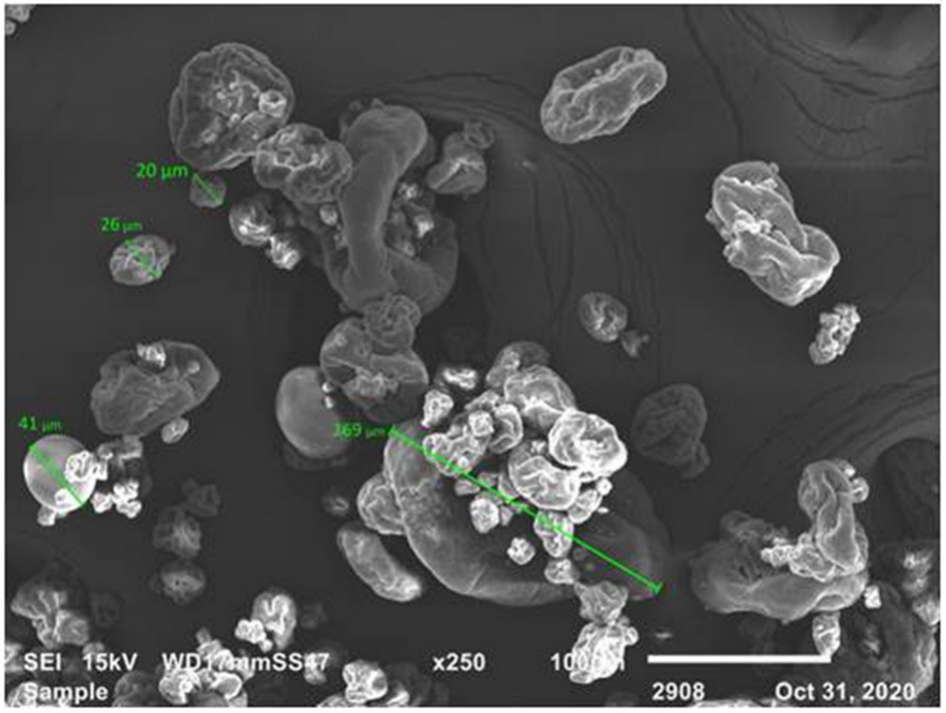
SEM micrograph of the yeast cell wall fraction (YCW). Scale bar = 100 μm.

The surface morphology and microstructure of the YCW preparation were assessed using SEM. An illustrative micrograph is shown in Figure 4. The image shows mostly β-glucan particles ranging from 20 to 169 μm in size, with some single particles. The majority of the unaggregated particles was approximately 27 ± 3 μm in size; however, the formation of aggregates between β-glucan particles was more noticeable. The SEM image also reveals the ridge-like nature of the β-glucan, with smooth surfaces and rolled-up edges. Finally, the microstructure of β-glucan particles was retained as indicated by their distinctive oval shape.

### *In vivo* Experiment

Data on the performance of experimental broilers are summarized in Table 3. At the end of week 1, no significant differences were noted in weight gain (WG) among the four dietary treatments. Nevertheless, by the end of week 2, WG was significantly reduced (*p* < 0.05) in chickens of the AF and AF + YCW groups, respectively. At the end of the trial (week 3), chickens receiving the AFB_1_-contaminated diet have a 28% reduction in WG. Moreover, WG was significantly improved (4.8%) in birds of the YCW treatment. Average daily feed intake (ADFI) was affected until 21 days of age. Furthermore, by the end of weeks 2 and 3, feed conversion ratio (FCR) was significantly affected in the AF and AF + YCW groups. Finally, the survival rate was as follows: 92% in the AF group, 96% in the AF + YCW group, and 100% in the control and YCW groups, respectively (Table 3). In general, the adverse effects in WG, ADFI, FCR, and survival rate—caused by AFB_1_–were significantly alleviated by the YCW treatment, which means that the YCW fraction offers reasonable protection against the harmful effects caused by AFB_1_.

**Table 3:** Production performance of experimental broiler chickens from 1 to 21 days of age.

**Attribute**	**Dietary treatments**				**SEM**	***p*-value**
	**Control**	**YCW**	**AF**	**AF + YCW**		
WG (g)						
1–7 days	110.7	112.2	100.6	105.9	1.47	0.257
7–14 days	200.4^a^	212.0^a^	135.3^c^	167.3^b^	8.07	0.001
14–21 days	269.0^b^	284.0^a^	181.2^d^	206.4^c^	15.41	0.007
1–21 days	580.1^b^	608.2^a^	417.1^d^	479.6^c^	12.69	0.005
Deviation from control (%)	0	4.8	−28.0	−17.3		
ADFI (g)						
1–7 days	18.98^a^	19.23^a^	16.38^b^	17.70^c^	0.19	0.009
7–14 days	36.07^b^	36.65^b^	33.43^c^	40.39^a^	0.11	0.001
14–21 days	51.11^a^	50.31^b^	49.44^c^	50.72^b^	0.17	0.009
FCR (feed:gain)						
1–7 days	1.20	1.20	1.14	1.17	0.01	0.207
7–14 days	1.26^a^	1.21^a^	1.73^b^	1.69^b^	0.02	0.002
14–21 days	1.33^b^	1.24^a^	1.91^d^	1.72^c^	0.04	0.003
MR (%)	0	0	8	4		

*Mean of five replicates of 10 chicks each per treatment (minus mortality). Means, within the same row, not sharing a common superscript differ significantly (Tukey test p < 0.05).*

*WG, weight gain; ADFI, average daily feed intake; FCR, feed conversion ratio; MR, mortality rate*.

Table 4 summarizes the serum biochemical results. AFB_1_ caused a significant decrement in total protein, albumin, glucose, and cholesterol concentrations. Compared to the control group, reductions of 29.5, 29.4, 17.6, and 38.8% in those constituents were observed in chickens of the AF group. Additionally, some indications of AFB_1_ toxicity were distinguished in the AF and AF + YCW groups' chickens by the serum AST activity level, which increased by 1.6- and 1.3-fold in comparison with the control group, respectively. On the contrary, no significant differences were observed in the ALT activity among all dietary treatments (Table 4). In general, the YCW preparation alleviates most of the biochemical parameters in the serum altered negatively due to AFB_1_.

**Table 4:** Selected serum biochemical profiles in broiler chickens at 21 days of age.

**Constituent**	**Dietary treatments**				**SEM**	***p*-value**
	**Control**	**YCW**	**AF**	**AF + YCW**		
Total protein (g/L)	27.5^a^	28.9^a^	19.4^c^	22.3^b^	1.65	0.002
Albumin (g/L)	11.9^a^	12.4^a^	8.4^b^	9.1^b^	0.03	0.001
Glucose (mg/dL)	471.2^a^	490.9^a^	388.1^b^	398.6^b^	20.9	0.009
Cholesterol (mg/dL)	148.8^a^	151.7^a^	91.1^b^	106.1^b^	3.75	0.005
AST (U/L)	140.7^c^	146.6^c^	227.5^a^	185.3^b^	7.38	0.007
ALT (U/L)	14.3	15.6	13.6	13.3	1.00	0.377

*Mean of five replicates of three chicks each per treatment (n = 15). Means, within the same row, not sharing a common superscript differ significantly (Tukey test p < 0.05).*

*AST, aspartate aminotransferase; ALT, alanine aminotransferase*.

Histomorphological parameters of the broiler's duodenum are summarized in Table 5. In general, villus height, villus area, crypt depth, and the number of goblet cells were significantly lower in chickens fed the AFB_1_-contaminated diet. The addition of the YCW preparation to the AFB_1_-contaminated diet increased villus height (1,211.1 vs. 964.0 μm); however, the value was significantly lower than the observed in the control group (1,435.7 μm). Furthermore, compared to the control group, chickens of the YCW group have higher villus height, villus area, crypt depth, and the number of goblet cells in villi. Villus width was not affected by any dietary treatment (Table 5 and Supplementary Figure 2).

**Table 5:** Histomorphological parameters of the broiler's duodenum at 21 days of age.

**Parameter**	**Dietary treatments**				**SEM**	***p*-value**
	**Control**	**YCW**	**AF**	**AF + YCW**		
Villus height (μm)	1,435.7^b^	1,749.5^a^	964.0^d^	1211.1^c^	55.9	0.003
Villus width (μm)	142.7	147.0	136.5	137.9	18.6	0.871
Villus area (mm^2^)	0.206^b^	0.253^a^	0.131^d^	0.163^c^	0.01	0.007
Crypt depth (μm)	161.9^b^	179.3^a^	124.4^d^	137.7^c^	8.1	0.041
Goblet cell (number/500 μm)	70.4^b^	96.0^a^	33.9^d^	62.1^c^	2.5	0.001

*Mean of 60 measurements per parameter. Means, within the same row, not sharing a common superscript differ significantly (Tukey test p < 0.05)*.

**Figure 5:**
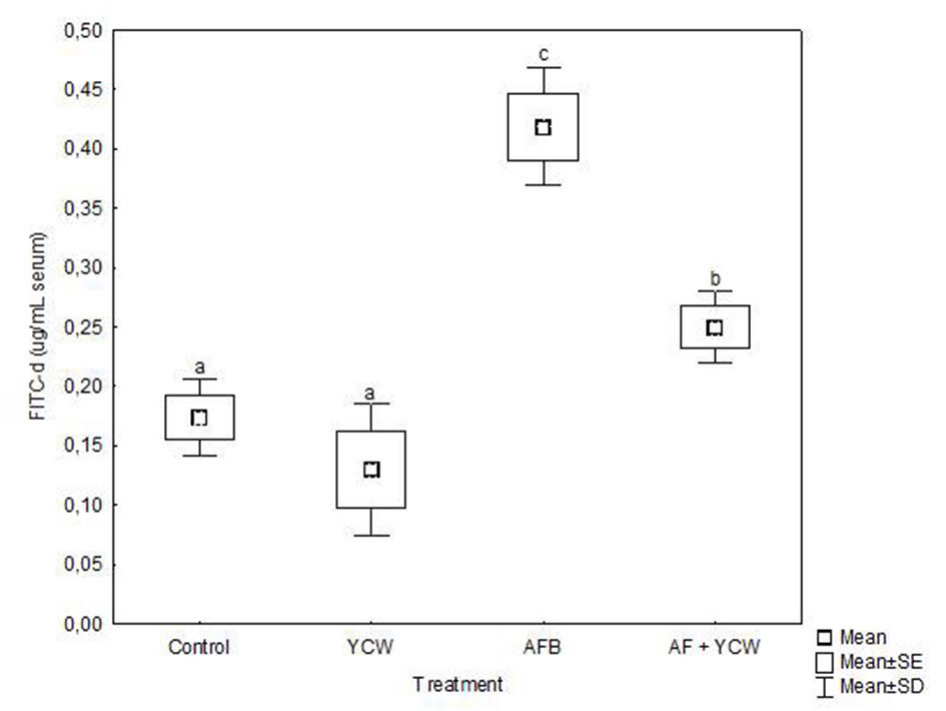
Serum fluorescein isothiocyanate dextran (FITC-d) levels in broiler chickens at 21 days of age. Mean of five replicates of three chicks each per treatment (*n* = 15). Boxes and whiskers not sharing a common superscript differ significantly (Tukey test *P* < 0.05).

Data on serum concentrations of FITC-d are shown in Figure 5. No significant differences were noted in serum levels of FITC-d between the control and YCW groups. However, a significant increment in serum levels of FITC-d was detected in birds fed the AFB_1_-contaminated diet, reaching values up to 0.42 ± 0.05 μg FITC-d/ml serum. Interestingly, YCW supplementation of the AFB_1_-contaminated diet significantly diminished the serum levels of FITC-d (40.5%). The AFB_1_-related toxic damage to the intestinal epithelium in broiler chickens was partially mitigated by incorporating the YCW product into the diet.

## Discussion

To confirm the effectiveness of the YCW preparation to bind AFB_1_, ζ-potential, pH_pzc_, FTIR, and SEM techniques were employed. The ζ-potential is a measurement of the charges on the surface of colloidal particles. Without a doubt, interface properties are significantly influenced by changes in pH, ionic strength, temperature, composition of the medium, among others. As a result, in this work, the YCW preparation was evaluated regarding its ζ-potential at pH values of the proventriculus (pH 2), crop (pH 5), and intestine (pH 7). It was observed that ζ-potential increased significantly with increasing pH reaching the maximum at pH 7 (Figure 1). This ζ-potential shift may be attributed to changes in cell wall charges. These results are in close agreement with Lavaisse et al. (20), who reported ζ-potential values of −6 and −16 mV for *Saccharomyces cerevisiae* cell wall at pH 3.5 and 5, respectively. On the other hand, the pH_pzc_ also provides useful information about particles' surface charge. The results indicate that the surface charge of the YCW product was zero at pH 3.09 (Figure 2). Thus, the YCW surface remained negatively charged when pH > pH_pzc_ > 3.09. As a result, the YCW preparation possesses significant AFB_1_-sorption uptakes in the crop (pH 5) and intestine (pH 7). On the contrary, in the proventriculus (pH 2), the contribution of electrostatic interactions would be drastically reduced, as the surface net charge of the YCW product is positive. In the present study, the high negative-charged surface of YCW particles (which remained mostly unchanged at pH values above the pH_pzc_) can be associated with their ability to remove AFB_1_ in some gastrointestinal tract compartments because of the enhancement of attractive forces between the AFB_1_ molecule and the YCW surface.

Infrared spectroscopy gives information at the molecular level, allowing the investigation of surface functional groups. In this research, the YCW preparation was further characterized to obtain information about the nature of the interaction between the functional groups present on the YCW surface and the AFB_1_ molecule. The FTIR spectrum of the YCW product is shown in Figure 3, profile A. In general, the YCW fraction exhibited higher quantities of functional groups associated with polysaccharides (3,279, 1,369, 1,202, 1,025, 887, and 810 cm^−1^), lipids (2,922, 2,849, and 1,711 cm^−1^), and proteins (3,279, 1,629, and 1,532 cm^−1^). These three main components have many different negatively charged functional groups responsible for the AFB_1_ adsorption (4, 10, 11). It has been reported that ~80% of the dry weight of the YCW product is made up of β-glucans and α-mannans (21). Besides, the outer layer of the *S. cerevisiae* cell wall is also composed of phosphomannans, which possess a net negative charge due to the presence of the phosphate group. However, the band associated with this functional group (located at approximately 1,070 cm^−1^) also appears in the polysaccharide absorbing region (1,200–750 cm^−1^); as a result, an in-depth band assignment is often complicated. However, glucan and mannan content in the YCW preparation was much higher than that of phosphates (Figure 3, profile B). These results corresponded well with transmittance intensities (or calculated areas) in the FTIR spectrum (Figure 3, profile A), showing the highest intensity/area in the polysaccharide region (R1), followed by lipid (R3) and protein (R2) regions, respectively.

The morphology and microstructure of the YCW product were examined by SEM. This material was determined to be a heterogeneous mixture of individual particles (27 ± 3 μm in size) and glucan particle aggregates up to 169 μm in length (Figure 4). Aggregation is a process in which materials joined together to generate themselves a mass or cluster, increasing or decreasing its porosity or density (12). In this research, the changes in cell wall negative charges due to changes in pH, the net negative surface charge above the pH_pzc_ 3.09, the higher quantities of negative charged functional groups on the YCW surface, and the formation of aggregates improved the efficiency and functionality of the YCW preparation resulting in a material with significant interaction with AFB_1_.

Aflatoxins cause important losses to the poultry industry due to reduced performance and health problems in the exposed birds. The results presented in Table 3, 4 show that AFB_1_ (500 ng AFB_1_/g of feed) significantly decreased WG and ADFI, increased FCR and MR, and induced negative changes in some biochemical parameters in broilers. These findings are following the results found by Hernández-Ramírez et al. (18). The authors reported that an experimental diet contaminated with 470 ng AFB_1_/g feed produced adverse effects on WG, FCR, MR, and serum biochemistry in broiler chickens at 21 days of age. Comparable results are also reported by other researchers (22–26). Moreover, the addition of the YCW product to the AFB_1_-contaminated diet did not alleviate the harmful effects caused by this mycotoxin. Still, it improved WG and FCR during the final stage of the experiment (14–21 days). These results confirm that the YCW preparation effect was undoubtedly due to its ability to adsorb AFB_1_, since one of the significant advantages of the glucan-base fractions in animal feeding is to interact with certain mycotoxins. In this context, several *in vitro* and *in vivo* reports have indicated that glucan-based binders prevent the toxic effects of different mycotoxins (6–8, 25, 27–32). In the current work, since the minimum manufacturers' recommended inclusion rate was utilized (0.05% w/w), the moderate efficacy of the YCW product to alleviate the adverse effects of AFB_1_ could be due to its saturation with the mycotoxin. Therefore, diets may need to be supplemented with YCW levels higher than 0.05% to achieve significant protective effects against 500 ng AFB_1_/g of feed.

The present research revealed positive effects of the YCW product on broiler performance (Table 3). These findings are in close agreement with earlier reports with broilers (33–39). The improved production performance in the YCW group might be related to an improvement in the apparent metabolizable energy intake (40), to the ability of the YCW preparation to stimulate broilers' immunity (41), and to the effects of YCW on disease resistance and gut health (42, 43). The last statement is more plausible because, in this research, the results of the YCW fraction on broiler performance may also be explained by its influence on duodenal histomorphology. In this context, the YCW preparation increased villus height, villus area, crypt depth, and the number of goblet cells in the villi of broiler chickens (Table 5). Similar results have been reported by different researchers (33, 35, 44–46).

Intestinal health is important for broiler performance. When it is impaired, gut histomorphology and gut barrier are damaged. In this sense, different *in vivo* studies have demonstrated that aflatoxins compromise the gastrointestinal tract's fundamental functions, including loss of barrier function (18, 47, 48). In the present study, intestinal permeability was significantly increased in the AF group (Figure 5), since birds presented a considerable increment in serum FITC-d concentration (up to 0.42 ± 0.05 μg/mL serum). However, the YCW fraction's addition to the AFB_1_-contaminated diet significantly diminished the serum levels of FITC-d (0.25 ± 0.03 μg/mL serum). These results confirm that the YCW fraction counteracted—to some extent—the AFB_1_-related toxic damage to the intestinal epithelium in broiler chickens. In this work, the insoluble property and structural conformation allowed β-glucans to adsorb AFB_1_ molecules mitigating their impact on the gastrointestinal tract. Unfortunately, the YCW fraction did not improve the intestinal epithelium's turnover and regeneration speed in birds of the AF + YCW group (Table 5). However, in addition to a significant increment in the villus height, a higher density of goblet cells was recorded in chickens of the AF + YCW group when compared with the AF group (62.1 vs. 32.9 cell/500 μm). These findings also support the idea that the YCW product alleviates the toxic effects of AFB_1_ on some histomorphological parameters of the duodenum. Furthermore, compared to the control group, a higher density of goblet cells was also recorded in chickens of the YCW group, suggesting that the YCW fraction can induce the proliferation of goblet cells (Table 5). Different authors have also reported an increased density of goblet cells in broilers-fed diets containing YCW (36, 49). Goblet cells are responsible for the synthesis, storage, and secretion of mucin—a high molecular weight glycoprotein—which represents the first line of defense of the small intestine against mycotoxins (50). In general, the quantity of mucin secreted is directly proportional to the number of goblet cells in villi. Consequently, in this research, an increase in the number of goblet cells in chickens of the AF + YCW group can be positively considered in view of mucus, protective effect against AFB_1_. Data on the effects of AFB_1_ on intestinal mucus production in broilers are still meager. However, Wu et al. (51) investigated the individual and combined effect of AFM_1_ (12 μM), ochratoxin A (20 μM), and zearalenone (100 μM) on the secretion of mucin-like glycoproteins in Caco-2/HT29-MTX cocultures. The authors found that double- and triple-mycotoxin combinations significantly reduced the expression of the highly glycosylated gel-forming mucins MUC2 and MUC5B. As a result, the researchers concluded that increased intestinal permeability is associated with a decrease in mucin secretion. To our knowledge, this is the first report on the effect of AFB_1_ (500 ng/g feed) on gut histomorphology and gut barrier in broiler chickens with low dietary supplementation of a commercial YCW product (0.5 g/kg). However, further *in vivo* studies will help improve our understanding of the link between AFB_1_, mucus production, and intestinal permeability in poultry.

## Data Availability Statement

The original contributions presented in the study are included in the article/Supplementary Material, further inquiries can be directed to the corresponding author/s.

## Ethics Statement

The animal study was reviewed and approved by Internal Committee for Care and Use of Experimental Animals of the Postgraduate Program in Animal Production and Health Sciences of the National Autonomous University of Mexico. Ethical approval code: CICUAE-C20_5.

## Author Contributions

JH-R, AV-D, and AM-A conceived and designed the experiment and wrote the paper. JH-R acquired, analyzed, and interpreted the data, and performed the statistical analysis. RM-G and GT-I took part in the discussion and helped in editing the manuscript. All authors contributed to the article and approved the submitted version.

## Conflict of Interest

The authors declare that the research was conducted in the absence of any commercial or financial relationships that could be construed as a potential conflict of interest.

## Publisher's Note

All claims expressed in this article are solely those of the authors and do not necessarily represent those of their affiliated organizations, or those of the publisher, the editors and the reviewers. Any product that may be evaluated in this article, or claim that may be made by its manufacturer, is not guaranteed or endorsed by the publisher.
