# Supplementary material for: Mitigation of AFB1-Related Toxic Damage to the Intestinal Epithelium in Broiler Chickens Consumed a Yeast Cell Wall Fraction
Source: Front Vet Sci. 2021 Jul 26;8:677965. doi: 10.3389/fvets.2021.677965 (PMC8350163; doi:10.3389/fvets.2021.677965)
Supplement: Supplementary file 1 [file Data_Sheet_1.docx]

Supplementary Material

**Supplementary Table 1**. Ingredient composition of the experimental diet.

| Ingredient | g/kg | Ingredient | g/kg |
| --- | --- | --- | --- |
| Maize | 574.5 | Threonine | 1.2 |
| Soybean meal | 346.6 | Choline chloride 60% | 2.0 |
| Vegetable oil | 34.5 | Vitamin premix ^1^ | 1.0 |
| Dicalcium phosphate | 18.6 | Mineral premix ^2^ | 1.0 |
| Calcium carbonate | 9.9 | Antioxidant ^3^ | 0.5 |
| Salt | 3.8 |  |  |
| DL-Methionine | 3.3 | Metabolizable energy (MJ/kg) | 12.7 |
| L-Lysine HCl | 3.1 | Crude protein | 221.5 |

^1^ Vitamin premix supplied the following per kg: vitamin A, 20,000,000 IU; vitamin D3, 6,000,000 IU; vitamin E, 75,000 IU; vitamin K3, 9 mg; thiamine, 3 mg; riboflavin, 8 mg, pantothenic acid, 18 mg; niacin, 60 mg; pyridoxine, 5 mg; folic acid, 2 mg; biotin, 0.2 mg; cyanocobalamin, 16 mg; and ascorbic acid, 200 mg. ^2^ Mineral premix supplied the following per kg; manganese, 120 mg; zinc, 100 mg; iron, 120 mg; copper, 10-15 mg; iodine, 0.7 mg; selenium, 0.4 mg; and cobalt, 0.2 mg. ^3^ Ethoxyquin.

**Supplementary Figure 1.** Representative UPLC-chromatogram from the analysis of the aflatoxin-contaminated feed. The retention times were 1.57 and 2.00 min for AFB_2_ and AFB_1_, respectively.

**Supplementary Figure 2.** Representative light micrographs of duodenum from chickens at 21 days of age fed diets: [A] Control, chickens fed an AFB_1_-free diet; [B] AF, chickens feed an AFB_1_-contaminated diet (500 ng AFB_1_/g); [C] YCW, chickens fed an AFB_1_-free diet + 0.05% YCW; and [D] AF+YCW, chickens feed an AFB_1_-contaminated diet (500 ng AFB1/g) + 0.05% YCW after H&E staining. Scale bar = 500 μm.


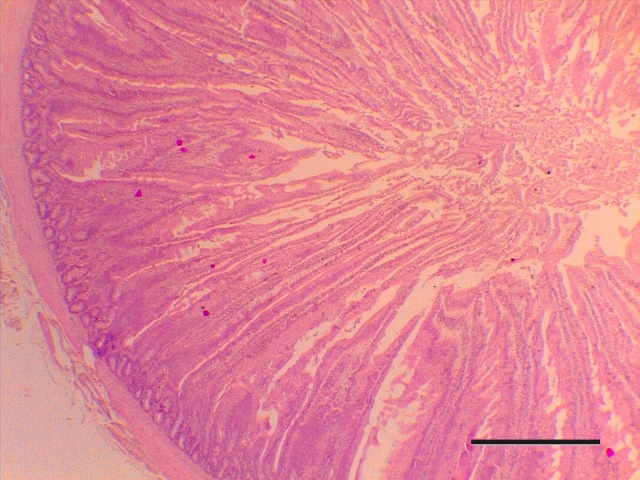


**A**


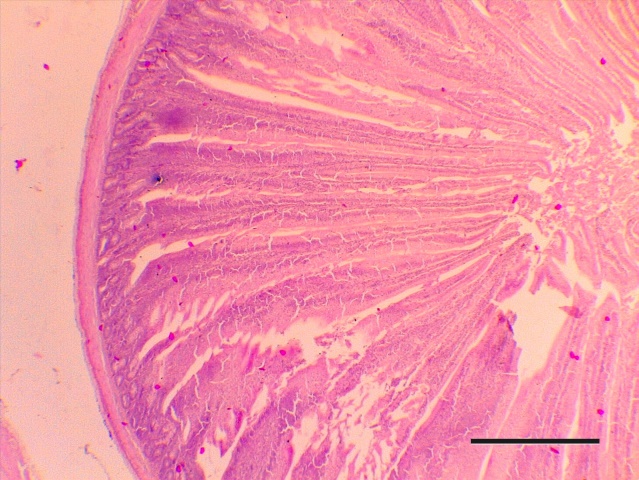


**C**


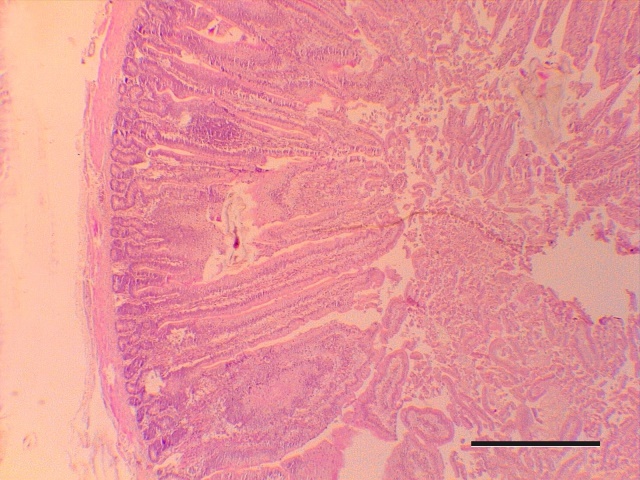


**B**


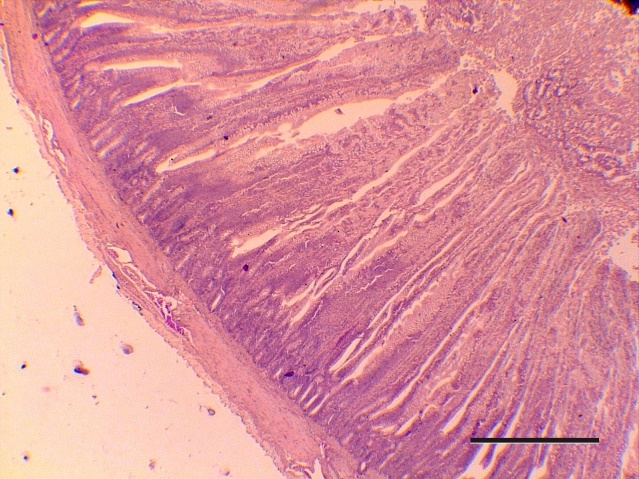


**D**
